# Concomitant 1q+ and t(4;14) influences disease characteristics, immune system, and prognosis in double-hit multiple myeloma

**DOI:** 10.1038/s41408-023-00943-2

**Published:** 2023-11-10

**Authors:** Michael Ozga, Qiuhong Zhao, Laila Huric, Cecelia Miller, Ashley Rosko, Abdullah Khan, Elvira Umyarova, Don Benson, Francesca Cottini

**Affiliations:** 1 The Ohio State University Comprehensive Cancer Center, Department of Internal Medicine, Division of Hematology, Columbus, OH USA; 2 The Ohio State University Comprehensive Cancer Center, Department of Pathology, Columbus, OH USA

**Keywords:** Translational research, Prognosis

Dear editor,

specific chromosomal abnormalities (CA) detected by Fluorescence in situ hybridization (FISH), including translocation t(4;14) and amplification or gain of chromosome 1q (1q+), confer inferior outcomes and shorter response to treatment [1, 2] in patients with Multiple Myeloma (MM). 1q+ can involve the whole long arm of chromosome 1 or only specific cytobands such as 1q21, 1q22, or 1q23.3 [3, 4], with 1q21 being the most established probe for detecting this CA by FISH. Whole-arm 1q gains confer inferior outcomes compared with focal gains underlying the importance of an accurate characterization of the 1q region [5]. 1q+ is often associated with t(4;14) as a cancer dependency [6]. The combined presence of 1q+ and t(4;14) defines a distinct subset of patients with double-hit (DH) MM, which is the focus of this report.

A retrospective chart review study (2021C0118) approved by the Ohio State University Institutional Review Board identified 243 unique MM patients who had available FISH data at diagnosis, and carried 1q21+ (probe CKS1B, Cytocell), 1q23+ (probe PBX1, Abbott), t(4;14), or 1q+ plus t(4;14) CA in the CD138 clone (Table S1). Most of them (209/243, 86.0%) were diagnosed after 2010. The median age at diagnosis was 61.0 years (range: 35–90 years), 53.9% were male, and 84% of patients were non-Hispanic White. 1q+ (1q21+ plus 1q23+) was present in 198/243 (81%) of patients, t(4;14) in 13/243 (5%) patients, and combined 1q+ and t(4;14) in 32/243 (13%) patients. Among all the patients with 1q+ CA (*n* = 230), 1q gains (3 copies) were more common than 1q amplifications (4 copies or more), and were present in 80.4% of patients (185/230). Other CAs (e.g., del(13q), del(17p), t(11;14) or *MAF* translocations) were equally balanced except for t(11;14), which was less common in patients with t(4;14) or DH MM, since t(11;14) and t(4;14) are often mutually exclusive [2].

The majority of the patients (184/243, 75.7%) were treated with bortezomib, lenalidomide, and dexamethasone (duplet or triplet regimen) as induction therapy. One hundred seventy (70%) patients underwent autologous stem cell transplant (ASCT) and 85.3% received maintenance therapy (145/170). 116/243 achieved complete response (CR) or very good partial response (VGPR) as best response (47.7%). Median follow up was 7.3 years (1.3–15.6 years). No statistical difference in progression-free survival (PFS) or overall survival (OS) from ASCT (Fig. S1A, B) was observed among these three cohorts.

Consistent with the literature [3, 7], t(4;14) occured almost exclusively in the primary clone (median: 78.5%), while 1q21+ or 1q23+ were present also at a subclonal level (1q21+ median: 48.7%; 1q23+ median: 53.95%) (Fig. S2A). No set cutoffs for these CAs are accepted in the literature, despite attempts of standardization [8]. To specifically evaluate the correlation of clone size to outcomes and disease characteristics, we established subgroupings with positivity cutoffs defined as 1q21+ or 1q23+ > 20%, t(4;14) > 30%, those who satisfy both cutoff criteria (DH), and those with clone sizes less than the prespecified cutoffs (“Low CA”).

Patients were then analyzed based on three strategies using the above cutoffs in the 1q21+ analysis, 1q23+ analysis, and 1q+ analysis (Fig. S2B, C and Tables S2, S3). We did not observe differences in terms of gender, race, MM subtype, staging, or laboratory findings at presentation in the three analyses. t(11;14) and del(13q) were differently distributed in the 1q21+ analysis (*p* = 0.03 and 0.01), but not in the 1q23+ or 1q+ analyses. Induction treatments among the cohorts were well balanced, with most of the patients achieving either CR or VGPR as best responses post-ASCT (Fig. S2D, E). No difference in the rates of ASCT or maintenance therapy was present among the different cohorts. The only observed differences were as follows: median age was significantly lower in patients with DH MM compared with the other groups (1q21 + analysis: *p* = 0.04, 1q23+ analysis: *p* = 0.04); and, in the 1q+ analysis, patients with combined 1q21+ and 1q23+ had less CR/VGPR compared with the other groups as best response (*p* = 0.08, Fig. S2F).

In the univariable analysis (UVA), patients with 1q21+ and t(4;14) at low levels (“Low CA” group) had statistically significant better outcomes in PFS and OS from ASCT compared to the corresponding DH group [PFS: HR = 0.52 (95% CI: 0.29–0.91), *p* = 0.02; Fig. 1A, and OS: HR = 0.39 (95% CI: 0.20–0.78), *p* = 0.008; Fig. 1B]. Median PFS was 4.4 years in the “Low CA” group compared with 2.1 years in the DH group, while median OS was 8.9 years in the “Low CA” group compared with 4.4 years in the DH group. This pattern in PFS and OS after ASCT was also observed in patients with 1q23+ and t(4;14) “Low CA” faring better compared to their DH group [PFS: HR = 0.46 (95% CI: 0.26–0.82), *p* = 0.009; Fig. 1C, and OS: HR = 0.33 (95% CI: 0.17–0.64), *p* = 0.001; Fig. 1D]. Patients with t(4;14) only (>30%) within the 1q23+ analysis also demonstrated longer PFS and OS after ASCT as compared to their DH group [PFS: HR = 0.44 (95% CI: 0.21–0.93), *p* = 0.03, and OS: HR = 0.41 (95% CI: 0.17–0.99), *p* = 0.04]. Median PFS was 3.0 years in the “Low CA” group compared with 1.9 years in the DH group, while median OS was 10 years in the “Low CA” group compared with 4.3 years in the DH group. After adjusting for age and 1q copy numbers in the multivariable analysis (MVA) presented in Table S4, the statistical difference in PFS and OS after ASCT remained significant in the 1q21+ analysis [“Low CA” versus DH PFS: HR = 0.52 (95% CI: 0.29–0.94), *p* = 0.029, and OS: HR = 0.38 (95% CI: 0.18–0.79), *p* = 0.009], and 1q23+ analysis [“Low CA” versus DH PFS: HR = 0.47 (95% CI: 0.25–0.87), *p* = 0.017, and OS: HR = 0.38 (95% CI: 0.18–0.78), *p* = 0.008]. Finally, independently of t(4;14), patients with combined 1q21+ and 1q23+ (Both group) had inferior PFS and OS from ASCT compared to patients with “Low/Neg” disease [Both versus “Low/Neg” PFS: HR = 1.93 (95% CI: 1.31–2.85), *p* = 0.001; Fig. 1E, and OS: HR = 2.04 (95% CI: 1.24–3.36), *p* = 0.006; Fig. 1F]. Median PFS was 4.1 years in the “Low/Neg” group compared with 1.6 years in the Both group, while median OS was 9.3 years in the “Low/Neg” group compared with 4.3 years in the Both group. Correcting for age and 1q copy numbers, these outcomes remained significant in the MVA [Both vs “Low/Neg” PFS: HR = 1.92 (95% CI: 1.21–3.04), *p* = 0.005, and OS: HR = 1.99 (95% CI: 1.08–3.64), *p* = 0.027, Table S4].

**Fig. 1:**
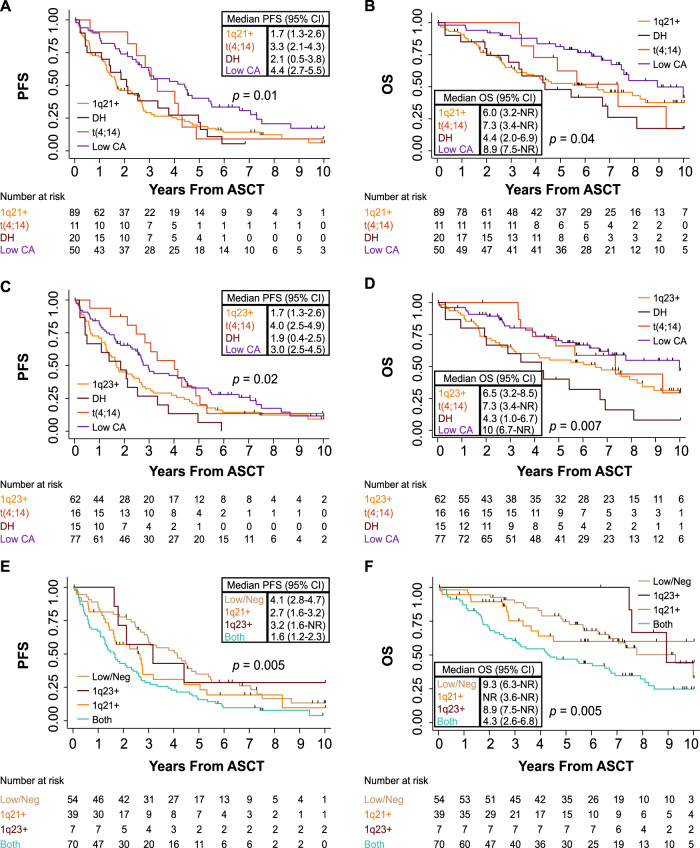
Kaplan-Meier curves for PFS and OS from ASCT. Log-rank *p* values and number at risk are reported for each graph in the panel. **A**, **B** PFS and OS for patients classified based on 1q21+. **C**, **D** PFS and OS for patients classified based on 1q23+. **E**, **F** PFS and OS for patients classified based on 1q+ abnormalities. *Abbreviations:* PFS Progression-free survival, OS overall survival, n number of patients, CI confidence interval, DH Double Hit, “Low CA” Low chromosomal abnormalities. NR not reached.

We recently studied the role of CD56 in MM, showing a correlation between CD56 expression and t(4;14) [9]; in the herein described cohort, we also observed that patients with DH had greater percentages of CD56-expressing cells compared with patients with “Low CA” (Fig. 2A) and higher CD56 mRNA expression (Fig. S3A). No significant differences in CD56 clone size were noted among the other groups or in the 1q+ analysis (Fig. S3B, C). Patients with DH MM also had more inhibitory CD94^+^NKG2A^+^ Natural Killer (NK) cells (Fig. 2B, C) and higher mRNA levels of HLA-E, the binding target of CD94/NKG2A (Fig. 2D). We also observed increased percentages of T regulatory cells (Fig. S4A) and CD4^+^CD25^-^LAG3^+^ cells (Fig. S4B, C) in patients with either t(4;14) only or DH MM.

**Fig. 2:**
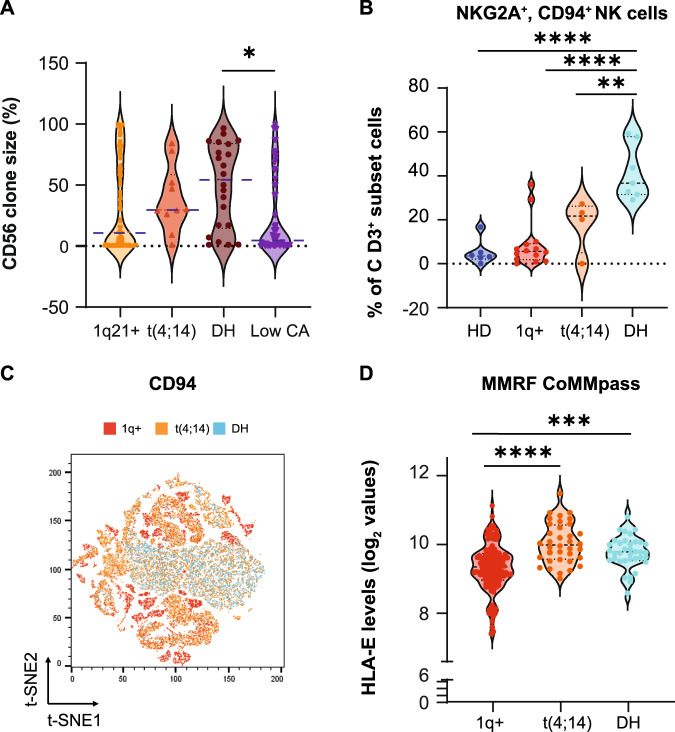
CD56 clone size and mRNA expression based on CA abnormalities. **A**. Violin plots showing the percentage of CD56-expressing clonal MM cells in patients with 1q21+ (*n* = 114), t(4;14) (*n* = 10), DH (*n* = 22), or “Low CA” (*n* = 58) MM in our database. ANOVA analysis with Bonferroni’s correction: *p* DH versus “Low CA” = 0.340 (*). ANOVA Summary *p* = 0.0461 (*). Blue dotted lines highlight the median value. If not reported, *p* are not significant. **B** Percentages of CD3^-^CD56^dim^CD16^+^CD94^+^NKG2A^+^ Natural Killer (NK) cells in healthy donors (HD, *n* = 7), patients with 1q+ (*n* = 13), t(4;14) (*n* = 4), or DH (*n* = 7) MM. ANOVA *p* < 0.0001. *p* HD versus DH < 0.0001 (****); *p* 1q+ versus DH < 0.0001 (****); *p* t(4:14) versus DH = 0.0075 (**). **C** t-SNE analysis combining CD94^+^/NKG2A^+^ cells in the three conditions. **D** FPKM levels of HLA-E in the CoMMpass MMRF database in patients with 1q+ (*n* = 133), t(4;14) (*n* = 36), and DH (*n* = 36). ANOVA *p* < 0.0001. *p* 1q+ versus DH = 0.0008 (***); *p* 1q+ versus t(4;14) < 0.0001 (****).

In summary, our study provides important insights into the field of 1q+/t(4;14) prognostication. First, since the effects on PFS and OS from ASCT are mainly evident in patients with large clones, our results suggest using a cutoff of positivity to properly categorize patients in the Revised International Staging System (ISS) [10], or in the R2-ISS score, which includes 1q+ in its algorithm [11]. Our results remained significant adjusting for 1q copy numbers, a known prognostic factor [1]. Second, the specific combination of genomic features is relevant. We describe the characteristics of a distinct subset of patients with DH, namely patients with combined 1q+ and t(4;14). These patients are younger than those with the corresponding single CA, have less t(11;14), and have inferior PFS and OS from ASCT. The noted difference in lenght of response to ASCT is likely related to both changes in the expression of MM genes and in immune populations. Herein we show that patients with DH MM have greater CD56 clones, higher CD56 and HLA-E mRNA levels, and more inhibitory CD94^+^NKG2A^+^ NK cells compared with patients with single CA. Matching CA, gene expression, and immunophenotyping data as in [12], is becoming more and more important to improve responses to MM-directed therapies [13, 14]. Finally, the location and involved cytobands in 1q+ also matter. Our study reports two important findings: 1. 1q23+ only also worsens the prognosis in patients with t(4;14); 2. larger gains of chromosome 1 (combined 1q21+ and 1q23+) confer inferior outcomes compared with focal 1q21+ or 1q23+ gains, confirming the recent data from Boyle et al. [5]. Using multiple probes on 1q chromosome as in [4] might hence allow to better stratification of patients with 1q+.

This study has some limitations. The cohort only included patients with the above CAs; therefore, we cannot define if the “Low CA” group performs similarly or worse than patients with standard risk MM. Similarly, flow cytometry data were limited to a subset of patients. Prior to FISH testing, all patient samples were immunomagnetically selected for CD138, however, due to limited specimen, purity was not assessed by a secondary method. Further research is needed to examine differences in plasma cell enrichment methodologies used by laboratories prior to FISH analysis to establish cutoffs [8]. Finally, while FISH analysis currently represents the gold-standard for clinical analysis, how these results will translate with the use of newer technologies which can examine copy numbers and structural variations at a higher resolution across the genome, such as next generation sequencing [6] and optical genome mapping [15], remains to be elucidated.

In conclusion, our study raises important questions in the field of 1q+/t(4;14) prognostication: 1. Is there a need to uniformily define CA cutoffs?; 2. Should we use multiple 1q probes?; 3. Are we ready to use these CAs as predictive markers of response? Answering these questions will improve estimation of risk and impact therapeutic choices in patients with MM.

### Supplementary information

Supplementary methods, tables, figures, and figure legends

## Data Availability

Data will be available upon request or are publicly available.

## References

[1] Schmidt TM, Barwick BG, Joseph N, Heffner LT, Hofmeister CC, Bernal L (2019). Gain of Chromosome 1q is associated with early progression in multiple myeloma patients treated with lenalidomide, bortezomib, and dexamethasone. Blood Cancer J.

[2] Walker BA, Leone PE, Chiecchio L, Dickens NJ, Jenner MW, Boyd KD (2010). A compendium of myeloma-associated chromosomal copy number abnormalities and their prognostic value. Blood..

[3] An G, Li Z, Tai YT, Acharya C, Li Q, Qin X (2015). The impact of clone size on the prognostic value of chromosome aberrations by fluorescence in situ hybridization in multiple myeloma. Clin Cancer Res.

[4] Zang M, Zou D, Yu Z, Li F, Yi S, Ai X (2015). Detection of recurrent cytogenetic aberrations in multiple myeloma: a comparison between MLPA and iFISH. Oncotarget..

[5] Boyle EM, Blaney P, Stoeckle JH, Wang Y, Ghamlouch H, Gagler D (2023). Multiomic mapping of acquired chromosome 1 copy number and structural variants to identify therapeutic vulnerabilities in multiple myeloma. Clin Cancer Res.

[6] Walker BA, Mavrommatis K, Wardell CP, Ashby TC, Bauer M, Davies FE (2018). Identification of novel mutational drivers reveals oncogene dependencies in multiple myeloma. Blood..

[7] Merz M, Jauch A, Hielscher T, Bochtler T, Schonland SO, Seckinger A (2018). Prognostic significance of cytogenetic heterogeneity in patients with newly diagnosed multiple myeloma. Blood Adv.

[8] Ross FM, Avet-Loiseau H, Ameye G, Gutierrez NC, Liebisch P, O’Connor S (2012). Report from the European Myeloma Network on interphase FISH in multiple myeloma and related disorders. Haematologica..

[9] Cottini F, Rodriguez J, Hughes T, Sharma N, Guo L, Lozanski G (2022). Redefining CD56 as a biomarker and therapeutic target in multiple myeloma. Mol Cancer Res.

[10] Palumbo A, Avet-Loiseau H, Oliva S, Lokhorst HM, Goldschmidt H, Rosinol L (2015). Revised international staging system for multiple myeloma: a report from international myeloma working group. J Clin Oncol.

[11] D’Agostino M, Cairns DA, Lahuerta JJ, Wester R, Bertsch U, Waage A (2022). Second revision of the international staging system (R2-ISS) for overall survival in multiple myeloma: a European Myeloma Network (EMN) report within the HARMONY project. J Clin Oncol.

[12] Steiger S, Lutz R, Prokoph N, Palit S, Tirier SM, Reichert P (2022). Bone marrow immune signatures in multiple myeloma are linked to tumor heterogeneity and treatment outcome. Blood..

[13] Friedrich MJ, Neri P, Kehl N, Michel J, Steiger S, Kilian M (2023). The pre-existing T cell landscape determines the response to bispecific T cell engagers in multiple myeloma patients. Cancer Cell.

[14] Martin T, Richardson PG, Facon T, Moreau P, Perrot A, Spicka I (2022). Primary outcomes by 1q21+ status for isatuximab-treated patients with relapsed/refractory multiple myeloma: subgroup analyses from ICARIA-MM and IKEMA. Haematologica..

[15] Kriegova E, Fillerova R, Minarik J, Savara J, Manakova J, Petrackova A (2021). Whole-genome optical mapping of bone-marrow myeloma cells reveals association of extramedullary multiple myeloma with chromosome 1 abnormalities. Sci Rep.

