# Supplementary material for: Concomitant 1q+ and t(4;14) influences disease characteristics, immune system, and prognosis in double-hit multiple myeloma
Source: Blood Cancer J. 2023 Nov 10;13(1):167. doi: 10.1038/s41408-023-00943-2 (PMC10638414; doi:10.1038/s41408-023-00943-2)
Supplement: Supplementary file 1 — Supplementary methods, tables, figures, and figure legends [file 41408_2023_943_MOESM1_ESM.pdf]

Concomitant 1q+ and t(4;14) influences disease characteristics, immune system, and prognosis in double-hit Multiple Myeloma

Authors:

Michael Ozga[1], Qiuhong Zhao[1], Laila Huric[1], Cecelia Miller[2], Ashley Rosko[1], Abdullah Khan[1], Elvira Umyarova[1], Don Benson[1], and Francesca Cottini[1].

[1] The Ohio State University Comprehensive Cancer Center, Department of Internal Medicine, Division of Hematology, Columbus, OH, USA

[2] The Ohio State University Comprehensive Cancer Center, Department of Pathology, Columbus, OH, USA

Correspondence:

Francesca Cottini, MD (lead contact)

Assistant Professor of Medicine

Division of Hematology

The Ohio State University, College of Medicine

385G Wiseman Hall | 400 W 12th Ave |

Columbus OH 43210-1240 USA

Email: francesca.cottini@osumc.edu

Supplementary Contents:

1. Extended Methods:

1.1. Patient characteristics
1.2. Definition of Responses and outcomes
1.3. Peripheral blood sample collection, isolation, and flow cytometry staining
1.4. CD56 flow cytometry analysis
1.5. RNA-sequencing Analysis
1.6. Statistical analysis

2. Supplementary Tables:

Supplementary Table S1. Patient characteristics of the full cohort of patients independently of chromosomal abnormality cutoffs.

Supplementary Table S2. Patient characteristics for 1q21+ or 1q23+ analyses.

Supplementary Table S3. Patient characteristics for 1q+ analysis.

Supplementary Table S4. Regression analysis for factors associated with progression-free survival (PFS) or Overall survival (OS) in the different groups.

3. Supplementary Figures:

3.1. Supplementary Figure S1. Outcomes of all patients with the defined chromosomal abnormalities.

3.2. Supplementary Figure S2. Study design and response rates.

3.3. Supplementary Figure S3. CD56 clone size and mRNA expression in the different subgroups.

3.4. Supplementary Figure S4. Immune profiling in selected patients with 1q+ only, t(4;14) only, or DH MM.

1. Extended methods:

1.1. Patient characteristics

This study was approved by the Ohio State University Institutional Review Board (2021C0118) after providing written informed consent for the Ohio State University MM registry (OSU-10115) in accordance with the Declaration of Helsinki. Specific cytogenetic abnormalities (CA) present at diagnosis included the presence of gain/amplification of chromosome arm 1q (1q21 (CKS1B, Cytocell) or 1q23 (PBX1, Abbott)), or t(4;14) (Abbott) as single abnormalities or in combination. CD138 enrichment was performed prior to FISH testing using the EasySep Human CD138 Positive Selection Cocktail. 200 interphases were analyzed for each probe, to provide sufficient cellularity. 1q copy numbers were used to define gains as the presence of 3 copies of 1q21+ or 1q23+ probes and amplifications as the presence of 4 or more copies of 1q21+ or 1q23+ probes by FISH.

1.2. Definition of Responses and outcomes

Response criteria were based on the International Myeloma Working Group synopsis and included complete responses (CR), very good partial responses (VGPR), partial responses (PR), stable disease (SD), or progressive disease (PD). Primary endpoints were progression-free survival (PFS) and overall survival (OS) after autologous stem cell transplant (ASCT). Kaplan Meier curves were used to calculate PFS and OS. Secondary endpoints included the identification of disease characteristics and their associations with PFS and OS. All tests were two-sided with p-values <0.05 considered statistically significant.

1.3. Peripheral blood sample collection, isolation, and flow cytometry staining

Peripheral blood samples from 7 healthy donors purchased from *Versiti, Inc* and 24 patients with active MM disease and the studied CA were separated by density gradient centrifugation over Ficoll-Paque, washed with centrifugation with room-temperature phosphate-buffered saline (PBS), and stained with the following flow cytometry fluorescent antibodies:

Antigen	Clone	Fluorophore	Supplier	Catalogue number
CD3	BW264/56	VioBlue	Miltenyi	170-081-046
CD4	RPA-T4 (RUO)	FITC	BD Bioscience	555346
CD8	BW135/80	APC	Miltenyi	130-113-154
LAG3/CD223	REA351	PE	Miltenyi	130-120-470
PD1/CD279	MIH4	PE	BD Bioscience	557946
CD28	15E8	PE	Miltenyi	130-126-172
CD25	M-A251	APC	BD Bioscience	555434
CD57	NK-1	FITC	BD Bioscience	555619
CD45RA	HI100	PE	BD Bioscience	561883
CD62L	145/15	VioBlue	Miltenyi	130-114-148
CD16	3G8	FITC	BD Bioscience	555406
CD56	REA196	APC Vio770	Miltenyi	130-114-548
CD56	B159	APC	BD Bioscience	555518
CD94	HP-3D9	PE	BD Bioscience	555889
NKG2A/CD159a	REA110	APC	Miltenyi	130-113-563
CD226	DX11	PE	BD Bioscience	559789
CD69	FN50	PE	Miltenyi	130-113-524
NKG2D/CD314	1D11	PE	BD Bioscience	557940

Cells were then incubated at room temperature for 20 minutes in a dark environment, re-washed with room-temperature PBS, and analyzed using an AttuneX machine. Post-acquisition analyses and t-distributed stochastic neighbor embedding (t-SNE) analyses were performed using FlowJo software.

1.4. CD56 flow cytometry analysis

The percentage of CD56-expressing CD138-positive, light-chain restricted MM cells (CD56 clone size) was obtained from the bone marrow aspirate at diagnosis. This staining was performed for clinical purposes in the Flow Cytometry Laboratory at The Ohio State University Wexner Medical Center, which is regulated under Clinical Laboratory

Improvement Amendments. CD56 clone size was correlated with the specific genomic abnormalities (1q21+ only, 1q23+ only, t(4;14), and Double Hit-DH).

1.5. RNA-sequencing Analysis

RNA-sequencing data were analyzed from MMRF CoMMpass. The MMRF data were generated as part of the Multiple Myeloma Research Foundation Personalized Medicine Initiatives. CD56 FKPM levels were correlated with the specific genomic abnormalities (1q+, t(4;14), and 1q+ and t(4;14)).

1.6. Statistical Analysis

Demographic and disease characteristics were summarized using medians and ranges for continuous variables, and frequencies and percentages for categorical variables. They were compared among cytogenetic groups using the Wilcoxon rank sum test, chi-square test or Fisher's exact test, respectively. PFS was calculated from ASCT to progression or death censoring those without progression at last followup and was estimated using the Kaplan-Meier method and compared between groups using the log-rank test. Overall survival was calculated from ASCT to death censoring those alive at last contact and compared using the log-rank test. Cox proportional hazard regression models were used to estimate the hazard ratios for risk of progression or death. The multivariable Cox model was built including all the variables significantly associated with PFS or OS in the univariable model. Analyses were performed using Stata version 17 (StataCorp, College Station, Texas), and all statistical tests were two-sided with statistical significance at 0.05.

2. Supplemental Tables:

Supplementary Table S1. Patient characteristics of the full cohort of patients independently of CA cutoffs.

	All (n = 243)	1q+ (n = 198)	t(4;14) (n = 13)	DH (n = 32)	*p*
**Age at diagnosis,** Median, Range	61.0 (35-90)	62.0 (35-90)	61.0 (40-86)	58.0 (40-71)	0.078
**Gender** (no, %)					
Female	112 (46.1)	92 (46.5)	3 (23.1)	17 (53.1)	0.18
Male	131 (53.9)	106 (53.5)	10 (76.9)	15 (46.9)	
**Race** (no, %)					
NHW	204 (84.0)	167 (84.3)	11 (84.6)	26 (81.3)	0.84
NHB	35 (14.4)	27 (13.6)	2 (15.4)	6 (18.8)	
Other	4 (1.6)	4 (2.0)	0	0	
**MM Subtype** (no, %)					
IgG	134 (55.1)	114 (57.6)	6 (46.2)	14 (43.8)	0.05
IgA	64 (26.3)	42 (21.2)	5 (38.5)	17 (53.1)	
IgD	7 (2.9)	7 (3.5)	0 (0)	0 (0)	
IgM	1 (0.4)	1 (0.5)	0 (0)	0 (0)	
Light chain disease	35 14.4)	32 (16.2)	2 (15.4)	1 (3.1)	
Non secretory	2 (0.8)	2 (1.0)	0 (0)	0	
**R-ISS stage** (no, %)					
I	33 (21.0)	33 (27.3)	0 (0)	0 (0)	0.003
II	91 (58.0)	65 (53.7)	6 (54.5)	20 (80)	
III	33 (21.0)	23 (19.0)	5 (45.5)	5 (20)	
**ISS stage** (no, %)					
I	69 (34.3)	55 (34.0)	3 (27.3)	11 (39.3)	0.48
II	70 (34.8)	55 (34.0)	3 (27.3)	12 (42.9)	
III	62 (30.8)	52 (32.1)	5 (45.5)	5 (17.9)	
**Hgb** (no, %)					
< 10 g/dL	83 (35.6)	63 (33.5)	6 (46.2)	14 (43.8)	0.38
**Serum creatinine** (no, %)					
≥ 2 mg/dL	49 (21.1)	39 (20.9)	5 (38.5)	5 (15.6)	0.23
**Calcium** (no, %)					
≥ 1 mg/dL	28 (12.5)	24 (13.4)	1 (7.7)	3 (9.4)	0.71
**Albumin** (no, %)					
< 3.5 g/dL	89 (41.4)	69 (40.1)	7 (58.3)	13 (41.9)	0.46
**LDH** (no, %)					
≥ 190 IU/L	55 (34.8)	44 (35.2)	4 (44.4)	7 (29.2)	0.70
**Beta-2 microglobulin** (no, %)					
< 3.5 mg/L	99 (48.5)	76 (46.1)	5 (45.5)	18 (64.3)	0.27
3.5-5.5 mg/L	45 (22.1)	39 (23.6)	1 (9.1)	5 (17.9)	
> 5.5 mg/L	60 (29.4)	50 (30.3)	5 (45.5)	5 (17.9)	
**t(11;14)** (no, %)					
Yes	44 (18.1)	42 (21.2)	1 (7.7)	1 (3.2)	0.03
No	199 (81.9)	156 (78.8)	12 (92.3)	31 (96.8)	
**del(13q)** (no, %)					
Yes	144 (59.3)	112 (56.6)	7 (53.9)	25 (78.2)	0.06
No	99 (40.7)	86 (43.4)	6 (46.1)	7 (21.8)	

**del(17p)** (no, %)					
Yes	31 (12.8)	26 (13.1)	1 (7.7)	4 (12.5)	0.85
No	212 (87.2)	172 (86.9)	12 (92.3)	28 (87.5)	
***MAF* translocations** (no, %)					
Yes	12 (4.9)	9 (4.6)	0	3 (9.4)	0.35
No	231 (95.1)	189 (95.4)	13 (100)	29 (90.6)	
**Copy Numbers of 1q** (no, %)	**(n = 230)**				
3 (gains)	185 (80.4)	159 (80.3)	0	26 (81.2)	<0.001
4 or more (amplifications)	45 (19.6)	39 (19.7)	0	6 (18.8)	
**Induction regimen** (no, %)					
CyBorD	29 (11.9)	21 (10.6)	3 (23.1)	5 (15.6)	0.90
VD	86 (35.4)	71 (35.9)	5 (38.5)	10 (31.3)	
RD	40 (16.5)	33 (16.7)	2 (15.4)	5 (15.6)	
VRD	58 (23.9)	47 (23.7)	2 (15.4)	9 (28.1)	
Other	30 (12.3)	26 (13.1)	1 (7.7)	3 (9.4)	
**Best response** (no, %)					
CR	30 (12.3)	20 (10.1)	2 (15.4)	8 (25.0)	0.28
VGPR	86 (35.4)	72 (36.4)	5 (38.5)	9 (28.1)	
PR	87 (35.8)	73 (36.9)	5 (38.5)	9 (28.1)	
SD	34 (14.0)	29 (14.6)	0	5 (15.6)	
PD	6 (2.5)	4 (2.0)	1 (7.7)	1 (3.1)	
**Response** (no, %)					
CR/VGPR	116 (47.7)	92 (46.5)	7 (53.8)	17 (53.1)	0.79
PR	87 (35.8)	73 (36.9)	5 (38.5)	9 (28.1)	
SD/PD	40 (16.5)	33 (16.7)	1 (7.7)	6 (18.8)	
**ASCT** (no, %)					
No	73 (30.0)	61 (30.8)	5 (38.5)	7 (21.9)	0.47
Yes	170 (70.0)	137 (69.2)	8 (61.5)	25 (78.1)	
**Maintenance** (no, %)					
No	25 (14.7)	18 (13.1)	2 (25.0)	5 (20.0)	0.47
Yes	145 (85.3)	119 (86.9)	6 (75.0)	20 (80.0)	

Abbreviations: DH, double Hit; *p*, p-value; NHW, non-hispanic white; NHB, non-hispanic black; MM, Multiple Myeloma; ISS, International staging system; R-ISS, Revised-ISS; Hgb, hemoglobin; LDH, lactate dehydrogenase; CA, chromosomal abnormalities; *MAF* translocations (t(14;16) or 14;20)); CyBorD, cyclophosphamide, bortezomib, dexamethasone; VD, bortezomib, dexamethasone; RD, lenalidomide, dexamethasone; VRD, bortezomib, lenalidomide, dexamethasone; CR, complete response; VGPR, very good partial response; PR, partial response; SD, stable disease; PD, progressive disease; ASCT, autologous stem cell transplant.

Supplementary Table S2. Patient characteristics for 1q21+ or 1q23+ analyses.

	1q21+ analysis					1q23+ analysis				
	**"Low CA" (n = 75)**	**1q21+ (n = 129)**	**t(4;14) (n = 14)**	**DH (n = 25)**	***p***	**"Low CA" (n = 118)**	**1q23+ (n = 86)**	**t(4;14) (n = 20)**	**DH (n = 19)**	***p***
**Age at diagnosis**, Median, Range	61.0 (38-86)	62.0 (35-90)	59.5 (40-71)	55 (40-71)	0.04	62 (35-86)	62 (39-90)	60 (40-71)	54 (45-70)	0.04
**Gender** (n, %)										
Female	31 (41.3)	63 (48.8)	2 (14.3)	16 (64)	0.02	51 (43.2)	43 (50)	8 (40)	10 (52.6)	0.67
Male	44 (58.7)	66 (51.2)	12 (85.7)	9 (36)		67 (56.8)	43 (50)	12 (60)	9 (47.4)	
**Race** (n, %)										
NHW	65 (86.7)	107 (83)	11 (78.6)	21(84)	0.92	95 (80.5)	77 (89.5)	16 (80)	16 (84.2)	0.38
NHB	9 (12)	19 (14.7)	3 (21.4)	4 (16)		19 (16.1)	9 (10.5)	4 (20)	3 (15.8)	
Other	1 (1.3)	3 (2.3)	0 (0)	0 (0)		4 (3.4)	0 (0)	0 (0)	0	
**MM Subtype** (n, %)										
IgG	42 (56)	73 (56.6)	8 (57.1)	11 (44)	0.18	73 (61.9)	42 (48.8)	9 (45)	10 (52.6)	0.35
IgA	14 (18.7)	33 (25.6)	4 (28.6)	13 (52)		22 (18.6)	25 (29.1)	9 (45)	8 (42.1)	
IgD	2 (2.7)	5 (3.8)	0 (0)	0 (0)		3 (2.5)	4 (4.7)	0 (0)	0 (0)	
IgM	1 (1.2)	0 (0)	0 (0)	0 (0)		1 (0.8)	0 (0)	0 (0)	0 (0)	
Light chain disease	14 (18.7)	18 (14)	2 (14.3)	1 (4)		17 (14.4)	15 (17.4)	2 (10)	1 (5.3)	
Non secretory	2 (2.7)	0 (0)	0 (0)	0 (0)		2 (1.7)	0 (0)	0 (0)	0 (0)	
**R-ISS stage** (n, %)										
I	12 (27.3)	21 (25.9)	0 (0)	0 (0)	0.07	19 (25.0)	14 (28.6)	0 (0)	0 (0)	0.08
II	23 (52.3)	45 (55.6)	7 (63.6)	16 (76.2)		43 (56.6)	25 (51.0)	11 (68.8)	12 (75.0)	
III	9 (20.5)	15 (18.5)	4 (36.4)	5 (23.8)		14 (18.4)	10 (20.4)	5 (31.2)	4 (25.0)	
**ISS stage** (n, %)										
I	23 (37.7)	35 (33)	3 (27.3)	8 (34.8)	0.93	29 (30.5)	29 (40.3)	4 (23.5)	7 (41.2)	0.64
II	19 (31.1)	37 (34.9)	4 (36.4)	10 (43.5)		32 (33.7)	24 (33.3)	8 (47.1)	6 (35.3)	
III	19 (31.1)	34 (32.1)	4 (36.4)	5 (21.7)		34 (35.8)	19 (26.4)	5 (29.4)	4 (23.5)	
**Hgb** (n,%)										
< 10 g/dL	20 (27.8)	45 (36.9)	6 (42.9)	12 (48)	0.26	38 (33.6)	27 (33.3)	8 (40)	10 (52.6)	0.40
**Creatinine** (n,%)										
≥ 2 mg/dL	16 (22.5)	25 (20.5)	4 (28.6)	4 (16)	0.81	26 (23.2)	15 (18.5)	4 (20)	4 (21.1)	0.89
**Calcium** (n, %)										
≥ 11 mg/dL	8 (11.8)	17 (14.5)	1 (7.1)	2 (8)	0.73	13 (11.8)	12 (16)	2 (10)	1 (5.3)	0.59
**Albumin** (n, %)										
< 3.5 g/dL	25 (37.9)	46 (41.4)	7 (53.8)	11 (44)	0.74	47 (46.1)	24 (32)	11 (57.9)	7 (36.8)	0.11
**LDH** (n, %)										
≥ 190 IU/L	17 (37)	28 (33.7)	3 (33.3)	7 (35)	0.99	27 (34.2)	18 (36)	5 (35.7)	5 (33.3)	1.00
**Beta-2 microglobulin** (n, %)										
< 3.5 mg/L	32 (51.6)	48 (44.4)	5 (45.5)	14 (60.9)	0.83	41 (42.3)	39 (53.4)	8 (47.1)	11 (64.7)	0.65
3.5-5.5 mg/L	12 (19.4)	27 (25)	2 (18.2)	4 (17.4)		24 (24.7)	15 (20.6)	4 (23.5)	2 (11.8)	
> 5.5 mg/L	18 (29)	33 (30.6)	4 (36.3)	5 (21.7)		32 (33)	19 (26)	5 (29.4)	4 (23.5)	
**t(11;14)** (n, %)										
Yes	19 (25.3)	24 (18.6)	0 (0)	1 (4)	0.03	29 (24.6)	14 (16.3)	0 (0)	1 (5.3)	0.2
No	56 (74.7)	105 (81.4)	14 (100)	24 (96)		89 (75.4)	72 (83.7)	20 (100)	18 (94.7)	
**del(13q)** (n, %)										
Yes	37 (49.3)	77 (59.7)	8 (57.1)	22 (88)	0.01	62 (52.5)	52 (60.5)	14 (70)	16 (84.2)	0.05
No	38 (50.7)	52 (40.3)	6 (42.9)	3 (12)		56 (47.5)	34 (39.5)	6 (30)	3 (15.8)	
**del(17p)** (n, %)										
Yes	7 (9.3)	20 (15.5)	2 (14.3)	3 (12)	0.65	15 (12.7)	12 (14)	2 (10)	3 (15.8)	0.95
No	68 (90.7)	109 (84.5)	12 (85.7)	22 (88)		103 (87.3)	74 (86)	18 (90)	16 (84.2)	
***MAF* translocations**										
(n, %) Yes	1 (1.3)	8 (6.2)	0 (0)	3 (12)	0.12	3 (2.5)	6 (7.0)	1 (5)	2 (10.5)	0.32
No	74 (98.7)	121 (93.8)	14 (100)	22 (88)		115 (97.5)	80 (93.0)	19 (95)	17 (89.5)	
**Copy Numbers of 1q**										
3 (gains)		107 (82.9)		21 (84)	<.001		69 (80.2)		15 (78.9)	<.001
4 or more (amplifications)		22 (17.1)		4 (16)			17 (19.8)		4 (21.1)	
**Induction regimen** (n, %)										
CyBorD	12 (16)	12 (9.3)	2 (14.3)	3 (12)	0.74	17 (14.4)	7 (8.1)	4 (20)	1 (5.3)	0.16
VD	29 (38.7)	43 (33.4)	5 (35.7)	9 (36)		41 (34.7)	31 (36)	7 (35)	7 (36.8)	
RD	10 (13.3)	23 (17.8)	3 (21.4)	4 (16)		12 (10.2)	21 (24.5)	2 (10)	5 (26.3)	
VRD	12 (16)	36 (27.9)	4 (28.6)	6 (24)		31 (26.3)	17 (19.8)	7 (35)	3 (15.8)	
Others	12 (16)	15 (11.6)	0 (0)	3 (12)		17 (14.4)	10 (11.6)	0 (0)	3 (15.8)	
**Best response** (n, %)										
CR	9 (12)	14 (10.9)	2 (14.3)	5 (20)	0.65	12 (10.1)	11 (12.8)	5 (25)	2 (10.5)	0.23
VGPR	30 (40)	43 (33.3)	4 (28.6)	9 (36)		50 (42.4)	23 (26.7)	8 (40)	5 (26.3)	
PR	23 (30.7)	52 (40.3)	6 (42.9)	6 (24)		39 (33.1)	36 (41.9)	6 (30)	6 (31.6)	

SD	13 (17.3)	16 (12.4)	1 (7.1)	4 (16)		15 (12.7)	14 (16.3)	0 (0)	5 (26.3)	
PD	0 (0)	4 (3.1)	1 (7.1)	1 (4)		2 (1.7)	2 (2.3)	1 (5)	1 (5.3)	
**Response** (n, %)										
CR/VGPR	39 (52)	57 (44.2)	6 (42.9)	14 (56)	0.70	62 (52.5)	34 (39.5)	13 (65)	7 (36.8)	0.14
PR	23 (30.7)	52 (40.3)	6 (42.9)	6 (24)		39 (33.1)	36 (41.9)	6 (30)	6 (31.6)	
SD/PD	13 (17.3)	20 (15.5)	2 (14.2)	5 (20)		17 (14.4)	16 (18.6)	1 (5)	6 (31.6)	
**ASCT** (n, %)										
No	25 (33.3)	40 (31)	3 (21.4)	5 (20)	0.54	41 (34.7)	24 (27.9)	4 (20)	4 (21.1)	0.37
Yes	50 (66.7)	89 (69)	11 (78.6)	20 (80)		77 (65.3)	62 (72.1)	16 (80)	15 (78.9)	
**Maintenance** (n, %)										
No	5 (10)	13 (14.6)	2 (18.2)	5 (25)	0.44	11 (14.3)	7 (11.3)	3 (18.7)	4 (26.7)	0.47
Yes	45 (90)	76 (85.4)	9 (81.8)	15 (75)		66 (85.7)	55 (88.7)	13 (81.3)	11 (73.3)	

Abbreviations: DH, double Hit; *p*, p-value; NHW, non-hispanic white; NHB, non-hispanic black; MM, Multiple Myeloma; ISS, International staging system; R-ISS, Revised-ISS; Hgb, hemoglobin; LDH, lactate dehydrogenase; CyBorD, cyclophosphamide, bortezomib, dexamethasone; VD, bortezomib, dexamethasone; RD, lenalidomide, dexamethasone; VRD, bortezomib, lenalidomide, dexamethasone; CR, complete response; VGPR, very good partial response; PR, partial response; SD, stable disease; PD, progressive disease; ASCT, autologous stem cell transplant.

Supplementary Table S3. Patient characteristics for 1q+ analysis

	"Low/Neg" (n = 79)	1q21+ only (n = 59)	1q23+ only (n = 10)	Both (n = 95)	*p*
**Age at diagnosis**, Median, Range	60.0 (38-86)	64.0 (35-82)	61.5 (55-78)	61.0 (39-90)	0.39
**Gender** (n, %)					
Female	29 (36.7)	30 (50.8)	4 (40)	49 (51.6)	0.20
Male	50 (63.3)	29 (49.2)	6 (60)	46 (48.4)	
**Race** (n, %)					
NHW	67 (84.8)	44 (74.6)	9 (90)	84 (88.4)	0.19
NHB	11 (13.9)	12 (20.3)	1 (10)	11 (11.6)	
Other	1 (1.3)	3 (5.1)	0 (0)	0 (0)	
**MM Subtype** (n, %)					
IgG	43 (54.4)	39 (66.1)	7 (70)	45 (47.4)	0.29
IgA	16 (20.3)	15 (25.4)	2 (20)	31 (32.6)	
IgD	2 (2.5)	1 (1.7)	0 (0)	4 (4.2)	
IgM	1 (1.3)	0 (0)	0 (0)	0 (0)	
Light chain disease	15 (19)	4 (6.8)	1 (10)	15 (15.8)	
Non secretory	2 (2.5)	0 (0)	0 (0)	0 (0)	
**R-ISS stage** (n, %)					
I	10 (19.6)	9 (22)	2 (50)	12 (19.7)	0.77
II	29 (56.9)	25 (61)	1 (25)	36 (59)	
III	12 (23.5)	7 (17)	1 (25)	13 (21.3)	
**ISS stage** (n, %)					
I	22 (34.4)	11 (22.9)	4 (50)	32 (39.5)	0.44
II	20 (31.3)	20 (41.7)	3 (37.5)	27 (33.3)	
III	22 (34.3)	17 (35.4)	1 (12.5)	22 (27.2)	
**Hgb** (n, %)					
< 10 g/dL	24 (31.6)	22 (38.6)	2 (20)	35 (38.9)	0.52
**Serum creatinine** (n, %)					
≥ 2 mg/dL	18 (24)	12 (21.1)	2 (20)	17 (18.9)	0.90
**Calcium** (n, %)					
≥ 11 mg/dL	8 (11)	7 (12.3)	1 (11.1)	12 (14.1)	0.94
**Albumin** (n, %)					
< 3.5 g/dL	29 (42)	29 (55.8)	3 (30)	28 (33.3)	0.07
**LDH** (n, %)					
≥ 190 IU/L	16 (32)	16 (37.2)	4 (80)	19 (31.7)	0.20
**Beta-2 microglobulin** (n, %)					
< 3.5 mg/L	31 (47.7)	18 (36.7)	6 (75)	44 (53.7)	0.40
3.5-5.5 mg/L	13 (20)	15 (30.6)	1 (12.5)	16 (19.5)	
> 5.5 mg/L	21 (32.3)	16 (32.7)	1 (12.5)	22 (26.8)	
**t(11;14)** (n, %)					
Yes	18 (22.8)	11 (18.7)	1 (10)	14 (14.7)	0.5
No	61 (77.2)	48 (81.3)	9 (90)	81 (85.3)	
**del(13q)** (n, %)					
Yes	41 (51.9)	35 (59.3)	4 (40)	64 (67.3)	0.12

No	38 (48.1)	24 (40.7)	6 (60)	31 (32.7)	
**del(17p)** (n, %)					
Yes	8 (10.1)	9 (15.3)	1 (10)	14 (14.7)	0.76
No	71 (89.9)	50 (84.7)	9 (90)	81 (85.3)	
***MAF* translocations** (n, %)					
Yes	1 (1.3)	3 (10.2)	0 (0)	8 (8.4)	0.16
No	78 (98.7)	56 (89.8)	10 (100)	87 (91.6)	
**Copy numbers of 1q** (n, %)					
3 (gains)		49 (83)	7 (70)	78 (82.1)	<0.001
4 or more (amplifications)		30 (17)	3 (30)	17 (17.9)	
**Induction regimen** (n, %)					
CyBorD	13 (16.5)	8 (13.6)	1 (10)	7 (7.4)	0.05
VD	31 (39.1)	17 (28.8)	3 (30)	35 (36.8)	
RD	10 (12.7)	4 (6.7)	3 (30)	23 (24.2)	
VRD	15 (19)	23 (39)	1 (10)	19 (20)	
Others	10 (12.7)	7 (11.9)	2 (20)	11 (11.6)	
**Best response** (n, %)					
CR	10 (12.7)	7 (11.9)	1 (10)	12 (12.6)	0.23
VGPR	29 (36.6)	29 (49.2)	5 (50)	23 (24.2)	
PR	27 (34.2)	18 (30.4)	2 (20)	40 (42.1)	
SD	12 (15.2)	3 (5.1)	2 (20)	17 (17.9)	
PD	1 (1.3)	2 (3.4)	0 (0)	3 (3.2)	
**Response** (n, %)					
CR/VGPR	39 (49.4)	36 (61)	6 (60)	35 (36.8)	0.08
PR	27 (34.2)	18 (30.5)	2 (20)	40 (42.1)	
SD/PD	13 (16.4)	5 (8.5)	2 (20)	20 (21.1)	
**ASCT** (n, %)					
No	25 (31.6)	20 (33.9)	3 (30)	25 (26.3)	0.75
Yes	54 (68.4)	39 (66.1)	7 (70)	70 (73.7)	
**Maintenance** (n, %)					
No	7 (13.0)	7 (17.9)	0 (0)	11 (15.7)	0.76
Yes	47 (87.0)	32 (82.1)	7 (100)	59 (84.3)	

Abbreviations: DH, double Hit; *p*, p-value; NHW, non-hispanic white; NHB, non-hispanic black; MM, Multiple Myeloma; ISS, International staging system, R-ISS, Revised-ISS; Hgb, hemoglobin; LDH, lactate dehydrogenase; CyBorD, cyclophosphamide, bortezomib, dexamethasone; VD, bortezomib, dexamethasone; RD, lenalidomide, dexamethasone; VRD, bortezomib, lenalidomide, dexamethasone; CR, complete response; VGPR, very good partial response; PR, partial response; SD, stable disease; PD, progressive disease; ASCT, autologous stem cell transplant.

Supplementary Table S4. Regression analysis for factors associated with progression-free survival (PFS) or Overall survival (OS) in the different groups.

	PFS			OS		
	**HR**	**95% CI**	***p***	**HR**	**95% CI**	***p***
**1q21+**	0.99	0.58-1.67	0.967	0.69	0.38-1.27	0.238
**t(4,14)**	0.69	0.27-1.76	0.44	0.68	0.23-2.01	0.489
**"Low CA"**	0.52	0.29-0.94	0.029	0.38	0.18-0.79	0.009
**Age**	0.99	0.97-1.02	0.611	1.02	0.99-1.05	0.246
**Gain(1q)**	0.96	0.47-1.94	0.905	1.04	0.43-2.52	0.935
**Amp(1q)**	1.06	0.49-2.29	0.889	1.45	0.56-3.76	0.45
**1q23+**	0.68	0.38-1.22	0.2	0.57	0.30-1.08	0.082
**t(4,14)**	0.44	0.19-1.03	0.058	0.54	0.19-1.59	0.265
**"Low CA"**	0.47	0.25-0.87	0.017	0.38	0.18-0.78	0.008
**Age**	0.99	0.97-1.02	0.557	1.02	0.99-1.05	0.32
**Gain(1q)**	1.01	0.63-1.62	0.976	1.22	0.60-2.46	0.584
**Amp(1q)**	1.03	0.56-1.89	0.973	1.75	0.77-4.01	0.183
**1q21+**	1.60	0.90-2.86	0.11	1.14	0.51-2.56	0.745
**1q23+**	0.69	0.25-1.90	0.471	0.52	0.14-1.93	0.328
**Both**	1.92	1.21-3.04	0.005	1.99	1.08-3.64	0.027
**Age**	0.99	0.97-1.02	0.53	1.01	0.98-1.05	0.347
**Gain(1q21+)**	0.79	0.41-1.52	0.476	0.75	0.32-1.76	0.508
**Amp(1q21+)**	1.01	0.34-3.03	0.981	1.14	0.29-4.54	0.848
**Gain(1q23+)**	1.26	0.75-2.12	0.376	1.24	0.61-2.52	0.557
**Amp(1q23+)**	0.98	0.33-2.88	0.972	1.12	0.29-4.33	0.874

Abbreviations: HR, Hazard Ratio; CI, Confidence interval; *p*, p-value; CA, chromosomal abnormalities.

3. Supplementary Figures:

Supplementary Figure S1. Outcomes of all patients with the defined chromosomal abnormalities. Log-rank *p* values and number at risk are reported for each graph in the panel. n = 170 patients who underwent ASCT. (**A, B**) PFS and OS from ASCT in patients with 1q+, t(4;14), and combined 1q+ and t(4;14), independently of cutoffs. *p* are not significant.

Supplementary Figure S2. **Study design and response rates. A.** Clone sizes of patients with 1q21+ (n = 216, median = 48.75), 1q23+ (n = 156, median = 53.95), and t(4;14) (n = 45, median = 78.5). **B.** Chromosome 1 probe representation. **C.** Schema of the reported analyses. 1q21+, 1q23+, and 1q+ analyses cohorts are shown. **(D-F).** Best responses are divided as complete responses/very good partial responses (CR/VGPR, light orange), partial responses (PR, orange), or stable disease, progressive disease (SD/PD, burgundy) in: **D.** patients with “Low CA”, 1q21+ only, t(4;14) only, or DH MM. *p* = 0.70. **E.** patients with “Low CA”, 1q23+ only, t(4;14) only, or DH MM. *p* = 0.14. **F.** patients with “Low/Neg”, 1q21+ only, 1q23+ only, and combined 1q21+ and 1q23+ (Both). *p* = 0.23.

Supplementary Figure S3. CD56 clone size and mRNA expression in the different subgroups. A. FPKM levels of CD56 in the CoMMpass MMRF database in patients with 1q+ (n = 133), t(4;14) (n = 36), and DH (n = 36) MM. *p* 1q+ versus t(4;14) < 0.0001 (****), *p* 1q+ versus DH < 0.0001 (****); *p* t(4;14) versus DH, Not significant. ANOVA Summary p < 0.0001. **B.** Violin plots showing the percentage of CD56-expressing clonal MM cells in patients with 1q23+ (n = 77), t(4;14) (n = 15), DH (n = 18), or “Low CA” (n = 95) MM in our database. **C**. Violin plots showing the percentage of CD56-expressing clonal MM cells

in patients with 1q21+ only (n = 51), 1q23+ only (n = 10), combined 1q21+ and 1q23+ (Both, n = 85), or no or low levels of 1q+ ("Low/neg", n = 58). *p* are not significant. In each panel, number of patients, median, and average values are reported below the plotted data. Blue dotted lines highlight the median values.

Supplementary Figure S4. **Immune profiling in selected patients with 1q+ only, t(4;14) only, or DH MM**. **A**. Percentages of $CD3^+CD4^+CD25^+$ T cells (T regulatory cells) in healthy donors, HD (n = 7), patients with 1q+ (n = 14), t(4;14) (n = 4), or DH (n = 7) MM. ANOVA $p = 0.0099$; *p* HD versus DH = 0.0125 (*); *p* 1q+ versus DH = 0.022 (*). **B.** Percentages of $CD3^+CD4^+LAG3^+$ T cells in HD (n = 7), patients with 1q+ (n = 13), t(4;14) (n = 4), or DH (n = 7) MM. ANOVA $p = 0.0002$. *p* 1q+ versus DH = 0.0021 (**), *p* 1q+ versus t(4;14) = 0.0234 (*); *p* HD versus DH = 0.0011 (**); *p* HD versus t(4;14) = 0.0097 (**). **C.** t-SNE analysis combining $LAG3^+$ cells in the three conditions.

Supplementary Figure S1

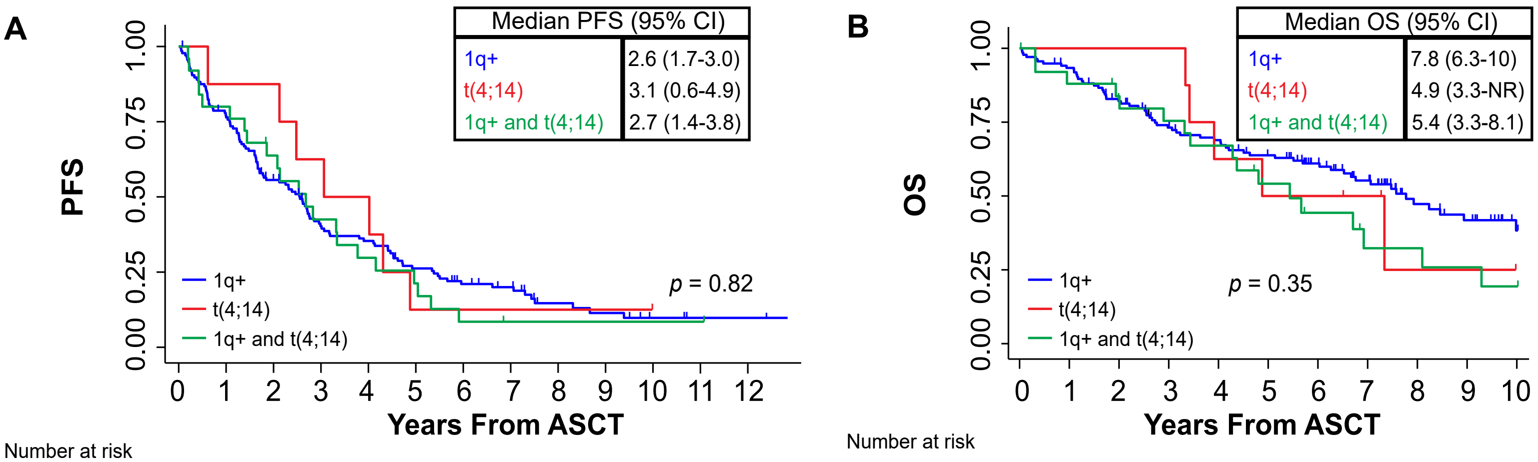

Number at risk (A)

1q+	137	103	72	49	43	31	22	18	9	7	3	1	1
t(4;14)	8	7	7	5	4	1	1	1	1	1	0	0	0
1q+ and t(4;14)	25	20	15	10	7	5	2	1	1	1	1	1	0

Number at risk (B)

1q+	137	125	106	87	81	72	56	45	27	22	11
t(4;14)	8	8	8	8	5	4	4	3	1	1	0
1q+ and t(4;14)	25	22	20	18	16	11	8	5	5	4	3

Supplementary Figure S1. **Outcomes of all patients with the defined chromosomal abnormalities.** Log-rank *p* values and number at risk are reported for each graph in the panel. n = 170 patients who underwent ASCT.
(A, B) PFS and OS from ASCT in patients with 1q+, t(4;14), and combined 1q+ and t(4;14), independently of cutoffs. *p* are not significant.

Supplementary Figure S2

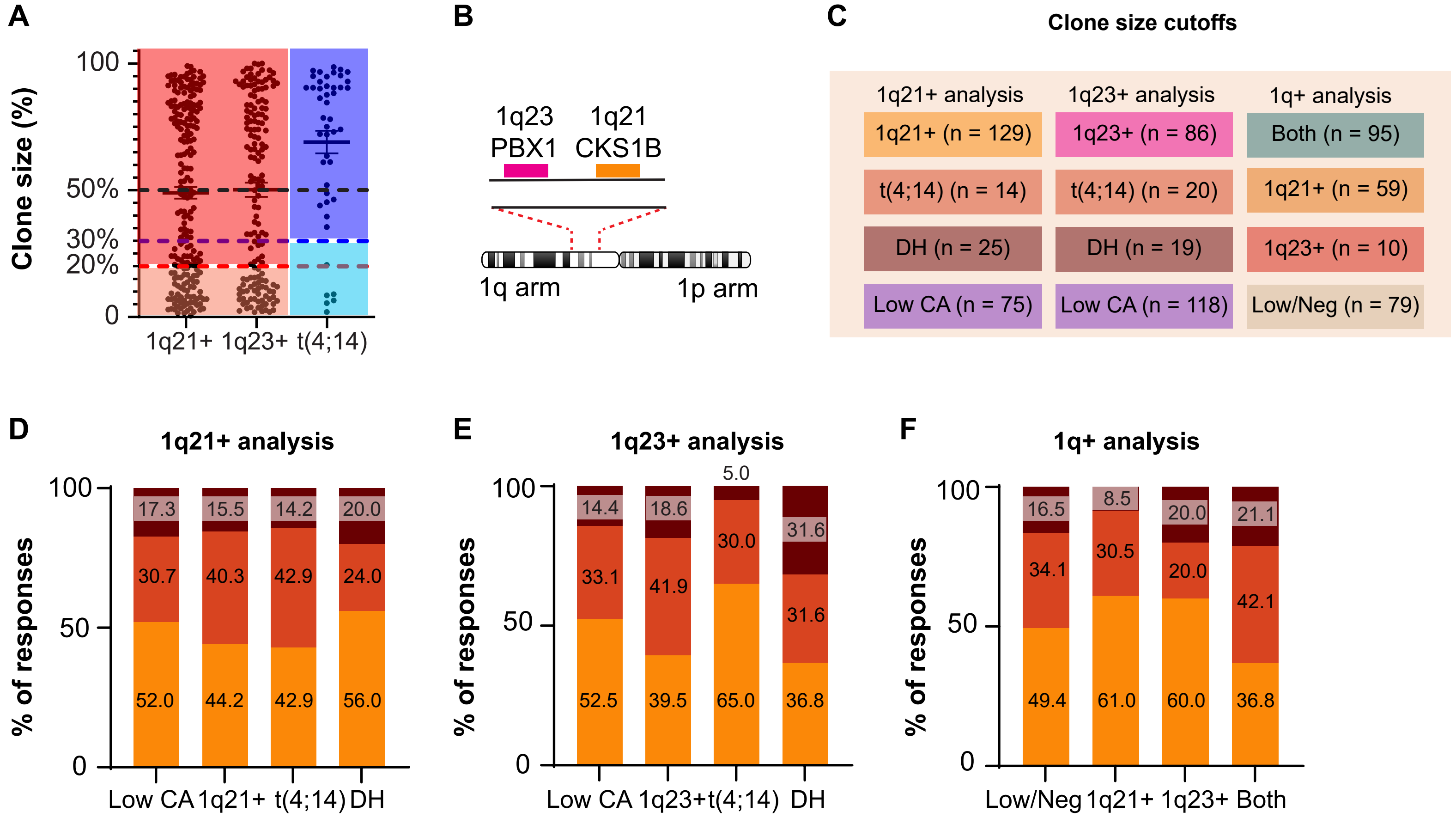

Supplementary Figure S2. Study design and response rates. A. Clone sizes of patients with 1q21+ (n = 216, median = 48.75), 1q23+ (n = 156, median = 53.95), and t(4;14) (n = 45, median = 78.5). **B.** Chromosome 1 probe representation. **C.** Schema of the reported analyses. 1q21+, 1q23+, and 1q+ analyses cohorts are shown. **(D-F).** Best responses are divided as complete responses/very good partial responses (CR/VGPR- light orange), partial responses (PR- orange), or stable disease, progressive disease (SD/PD, burgundy) in: **D.** patients with "Low CA", 1q21+ only, t(4;14) only, or DH MM. *p* = 0.70. **E.** patients with "Low CA", 1q23+ only, t(4;14) only, or DH MM. *p* = 0.14. **F.** in patients with "Low/Neg", 1q21+ only, 1q23+ only, and combined 1q21+ and 1q23+ (Both). *p* = 0.23.

Supplementary Figure S3

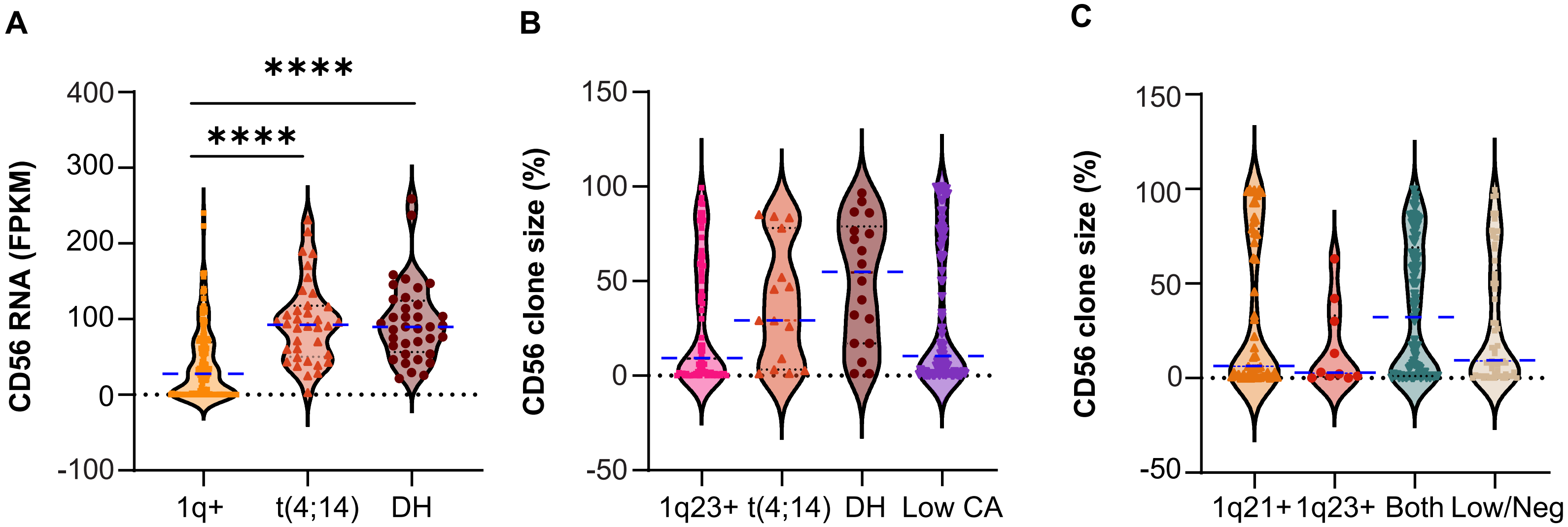

	1q+	t(4;14)	DH
n:	133	36	36
Median:	27.55	92.47	89.1
Average:	41.26	96.04	96.29

	1q23+	t(4;14)	DH	Low CA
n:	77	15	18	95
Median:	9	29.20	54.5	8.00
Average:	29.58	38.5	50.26	30.51

	1q21+	1q23+	Both	Low/Neg
n:	51	10	85	58
Median:	6.3	2.6	32	9.050
Average:	34.96	15.52	36.95	29.14

Supplementary Figure S3. CD56 clone size and mRNA expression in the different subgroups.
A. FPKM levels of CD56 in the CoMMpass MMRF database in patients with 1q+ (n = 133), t(4;14) (n = 36), and DH (n = 36) MM. *p* 1q+ versus t(4;14) < 0.0001 (****), *p* 1q+ versus DH < 0.0001 (****); *p* t(4;14) versus DH, Not significant. ANOVA Summary *p* < 0.0001. **B.** Violin plots showing the percentage of CD56-expressing clonal MM cells in patients with 1q23+ (n = 77), t(4;14) (n = 15), DH (n = 18), or “Low CA” (n = 95) MM in our database. **C.** Violin plots showing the percentage of CD56-expressing clonal MM cells in patients with 1q21+ only (n = 51), 1q23+ only (n = 10), 1q21+ and 1q23+ (Both, n = 85), or no or low levels of 1q+ (Low/Neg, n = 58). *p* are not significant. In each panel, number of patients, median, and average values are reported below the plotted data. Blue dotted lines highlight the median values.

Supplementary Figure S4

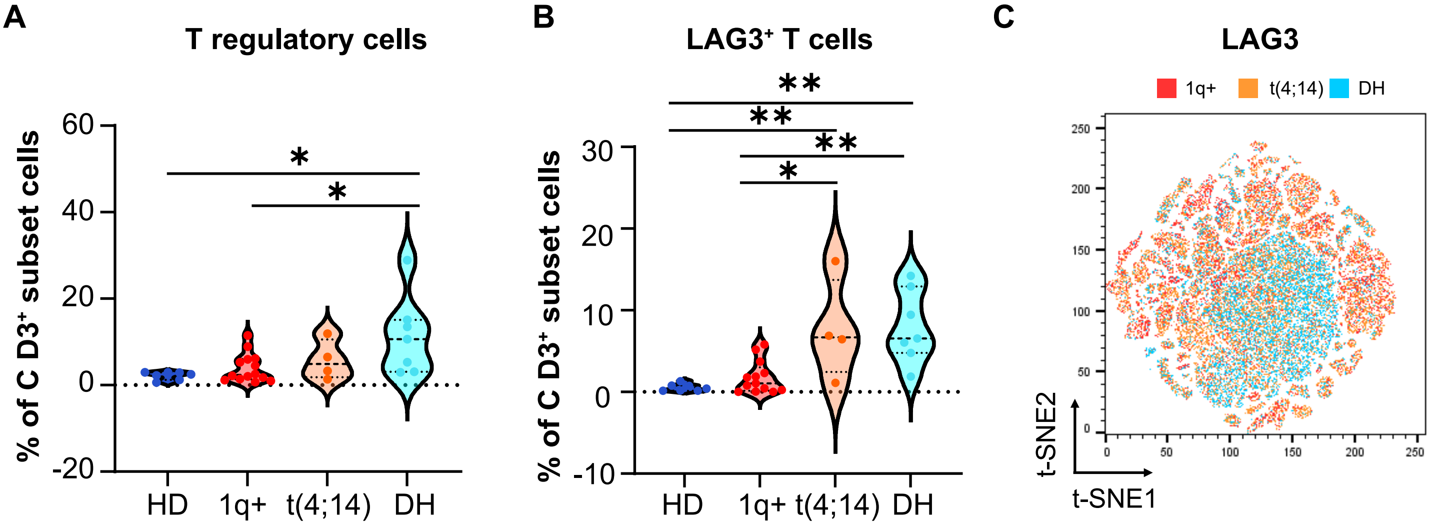

Supplementary Figure S4. Immune profiling in selected patients with 1q+ only, t(4;14) only, or DH MM. **A.** Percentages of $CD3^{+}CD4^{+}CD25^{+}$ T cells (T regulatory cells) in healthy donors, HD (n = 7), patients with 1q+ (n = 14), t(4;14) (n = 4), or DH (n = 7) MM. ANOVA $p = 0.0099$; p HD versus DH = 0.0125 (*); p 1q+ versus DH = 0.022 (*). **B.** Percentages of $CD3^{+}CD4^{+}LAG3^{+}$ T cells in HD (n = 7), patients with 1q+ (n = 13), t(4;14) (n = 4), or DH (n = 7) MM. ANOVA $p = 0.0002$. p 1q+ versus DH = 0.0021 (**), p 1q+ versus t(4;14) = 0.0234 (*); p HD versus DH = 0.0011 (**); p HD versus t(4;14) = 0.0097 (**). **C.** t-SNE analysis combining $LAG3^{+}$ cells in the three conditions.
